# P-1211. Effectiveness of iMIpenem-Relebactam for multidrug-resistant Pseudomonas AeruGinosa in pnEumonia and bloodstream infections in the United States (MIRAGE)

**DOI:** 10.1093/ofid/ofaf695.1404

**Published:** 2026-01-11

**Authors:** Walaiporn Wangchinda, Janet Y Wu, jason M Pogue, Lilian M Abbo, Renee Ackley, Patricia Bartley, mayan Gilboa, Jeffrey Harrington, Rupal K Jaffa, Megan Klatt, Ellen G Kline, Ryan C Kubat, Alexander J Lepak, Erin K McCreary, William R Miller, Jeffrey C Pearson, Sunish Shah, Truc Cecilia Tran, Ana Vega, Emre Yucel, Ryan K Shields

**Affiliations:** Siriraj Hospital Mahidol University, -, Krung Thep, Thailand; Cleeveland Clinic, Cleveland, Ohio; University of Michigan, College of Pharmacy, Ann Arbor, MI; University of Miami School of Medicine, Miami, Florida; Atrium Health, Charlotte, North Carolina; Cleveland Clinic, Cleveland, OH; Sheba Medical Center, Aventura, Florida; University of Michigan Health, Ann Arbor, MI; Atrium Health, Charlotte, North Carolina; The University of Kansas Health System, KS; University of Pittsburgh, Pittsburgh, Pennsylvania; University of Kansas, Kansas City, Kansas; University of Wisconsin School of Medicine and Public Health, Madison, WI; University of Pittsburgh Medical Center, Pittsburgh, PA; Houston Methodist Research Institute, Houston, TX; Brigham and Women's Hospital, Boston, MA; Antibiotic Management Program, UPMC Presbyterian Hospital, Pittsburgh, PA, Pittsburgh, Pennsylvania; Houston Methodist Research Institute, Houston, TX; Jackson Memorial Hospital, Miami, Florida; Merck & Co., Inc., North Wales, PA; University of Pittsburgh, Pittsburgh, Pennsylvania

## Abstract

**Background:**

Imipenem/relebactam (I/R) demonstrates potent *in vitro* activity against multidrug-resistant (MDR) *Pseudomonas aeruginosa*. The objective of this study was to evaluate the effectiveness of I/R for treatment of MDR *P. aeruginosa* infections across the U.S.

**Table 1.**
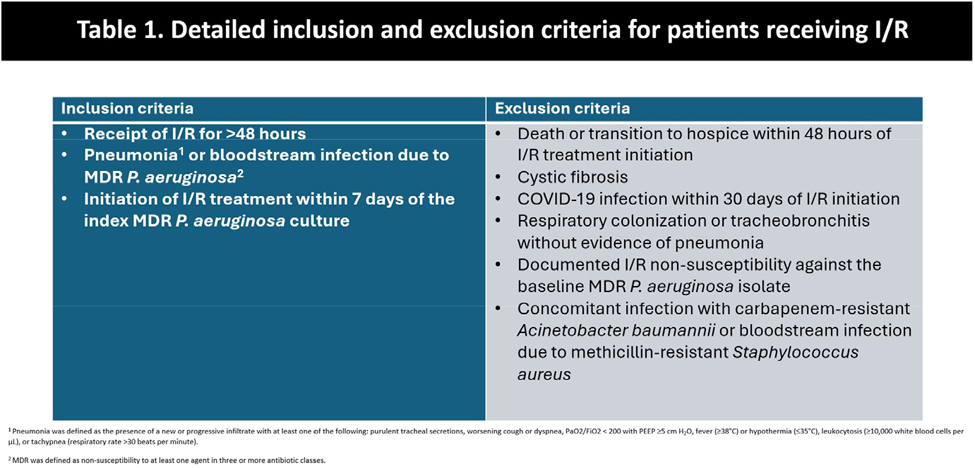
Detailed inclusion and exclusion criteria for patients receiving I/R 1 Pneumonia was defined as the presence of a new or progressive infiltrate with at least one of the following: purulent tracheal secretions, worsening cough or dyspnea, PaO2/FiO2 < 200 with PEEP ≥5 cm H2O, fever (≥38°C) or hypothermia (≤35°C), leukocytosis (≥10,000 white blood cells per µL), or tachypnea (respiratory rate >30 beats per minute). 2 MDR was defined as non-susceptibility to at least one agent in three or more antibiotic classes.

**Table 2.**
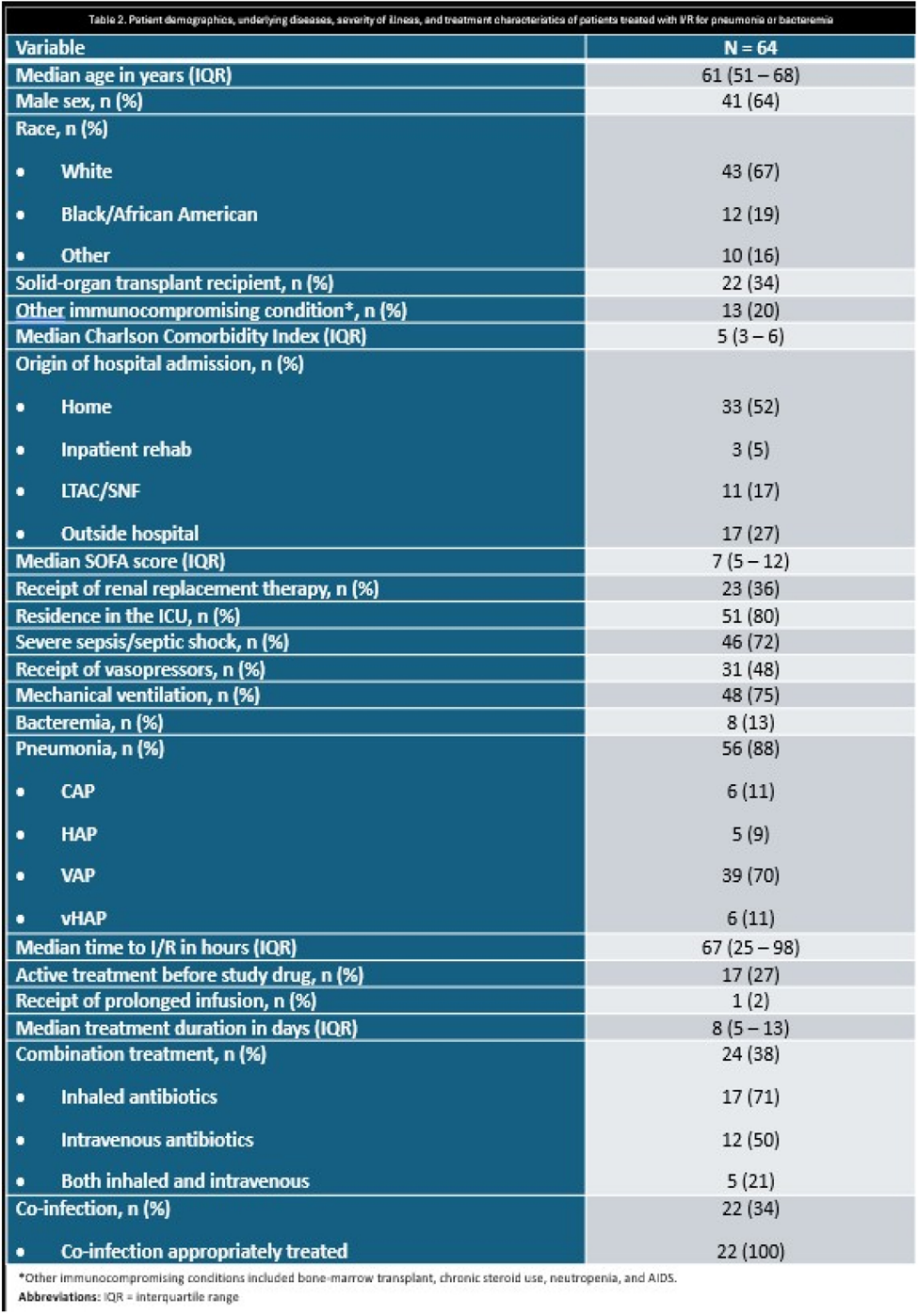
Patient demographics, underlying diseases, severity of illness, and treatment characteristics of patients treated with I/R for pneumonia or bacteremia. *Other immunocompromising conditions included bone-marrow transplant, chronic steroid use, neutropenia, and AIDS. Abbreviations: IQR = interquartile range

**Methods:**

This was a retrospective, multicenter, observational study of I/R for MDR *P. aeruginosa* pneumonia and bacteremia. Patients were included if they received I/R for >48h initiated within 7 days of the index MDR *P. aeruginosa* culture (Table 1). Clinical success was defined as survival, resolution of signs and symptoms of infection, completion of the intended treatment course, and the absence of a recurrent infection due to MDR *P. aeruginosa*. I/R susceptibility was determined by site-level microbiology labs; non-susceptibility was defined by the Clinical and Laboratory Standards Institute (CLSI) criteria.

**Table 3.**
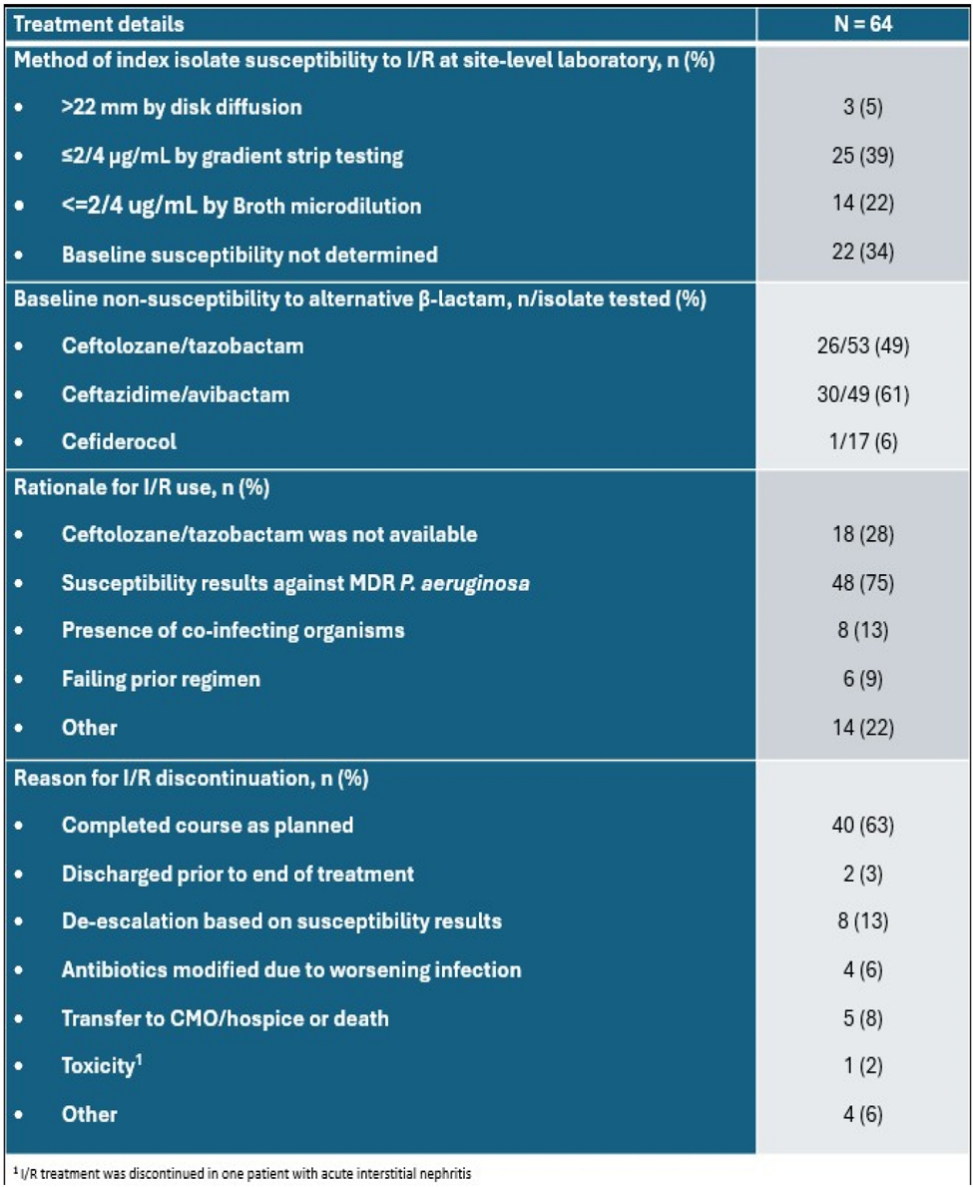
Real-world characteristics of I/R use in pneumonia and bloodstream infections. 1 I/R treatment was discontinued in one patient with acute interstitial nephritis

**Table 4.**
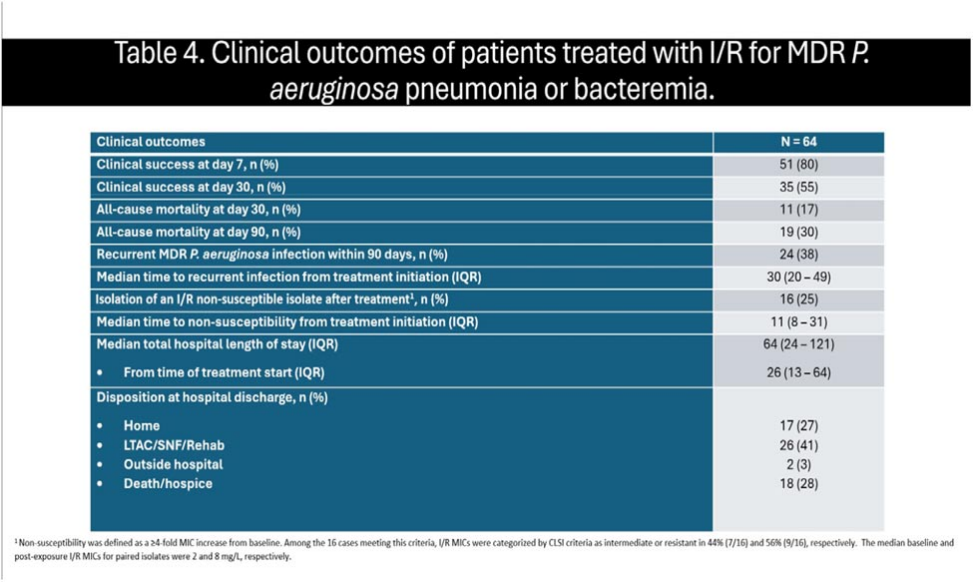
Clinical outcomes of patients treated with I/R for MDR P. aeruginosa pneumonia or bacteremia 1 Non-susceptibility was defined as a categorical change from susceptible to non-susceptible as defined by CLSI interpretive criteria. Among the 16 cases meeting this criteria, non-susceptibility was identified by gradient strip testing and broth microdilution in 25% and 75%, respectively. The median I/R MICs for isolates categorized as susceptible and non-susceptible were 2 and 8 mg/L, respectively.

**Results:**

64 patients from 10 centers were included (Table 2); patients from 6 additional centers were screened and did not meet inclusion criteria. The overall cohort was critically-ill; 80%, 75%, and 48% were in the intensive care unit, receiving mechanical ventilation, and on vasopressors, respectively. The median (interquartile range; IQR) SOFA score was 7 (5 – 12). 53% received treatment with another new β-lactam for MDR *P. aeruginosa* infections prior to I/R. The median time to I/R initiation was 67 hours. I/R treatment was primarily prescribed based on susceptibility results in 75% of patients, including resistance to other novel β-lactam agents (Table 3). 63% of patients completed the intended I/R treatment course as planned. At day 7 and 30, 80% and 55% met criteria for clinical success, respectively (Table 4). The overall 30- and 90-day mortality rates were 17% and 30%, respectively. Recurrent infections were documented in 38% of patients within 90 days.

**Conclusion:**

In this critically-ill patient population we found that I/R was often used following treatment with other novel β-lactams. Clinical outcomes were generally comparable to those previously reported in similar real-world studies for other novel β-lactam agents suggesting that I/R plays a role in treatment of MDR *P. aeruginosa* infections, particularly when other agents are not available or test resistant.

**Disclosures:**

jason M. Pogue, PharmD, Entasis: Advisor/Consultant|Entasis: Grant/Research Support|GlaxoSmithKline: Advisor/Consultant|Melinta: Grant/Research Support|Merck: Advisor/Consultant|Merck: Grant/Research Support|Shionogi: Advisor/Consultant|Shionogi: Grant/Research Support Alexander J. Lepak, MD, FIDSA, BioMerieux: Grant/Research Support William R. Miller, M.D., Merck: Grant/Research Support|UpToDate: Royalties, topic author Jeffrey C. Pearson, PharmD, InflaRx Pharmaceuticals, Inc.: Advisor/Consultant Emre Yucel, PhD, Merck & Co., Ltd: Stocks/Bonds (Public Company)

